# A method for analyzing programmed cell death in xylem development by flow cytometry

**DOI:** 10.3389/fpls.2023.1196618

**Published:** 2023-06-09

**Authors:** Ying-Li Liu, Ying-Hua Guo, Xue-Qin Song, Meng-Xuan Hu, Shu-Tang Zhao

**Affiliations:** ^1^ State Key Laboratory of Tree Genetics and Breeding, Chinese Academy of Forestry, Beijing, China; ^2^ National Center for Protein Sciences at Peking University, Beijing, China; ^3^ State Key Laboratory of Tree Genetics and Breeding, Research Institute of Forestry, Chinese Academy of Forestry, Beijing, China

**Keywords:** PCD, xylem development, FACS, gene expression, woody plants

## Abstract

Programmed cell death (PCD) is a genetically regulated developmental process leading to the death of specific types of plant cells, which plays important roles in plant development and growth such as wood formation. However, an efficient method needs to be established to study PCD in woody plants. Flow cytometry is widely utilized to evaluate apoptosis in mammalian cells, while it is rarely used to detect PCD in plants, especially in woody plants. Here, we reported that the xylem cell protoplasts from poplar stem were stained with a combination of fluorescein annexin V-FITC and propidium iodide (PI) and then sorted by flow cytometry. As expected, living cells (annexin V-FITC negative/PI negative), early PCD cells (annexin V-FITC positive/PI negative), and late PCD cells (annexin V-FITC positive/PI positive) could be finely distinguished through this method and then subjected for quantitative analysis. The expression of cell-type- and developmental stages-specific marker genes was consistent with the cell morphological observation. Therefore, the newly developed fluorescence-activated cell sorting (FACS) method can be used to study PCD in woody plants, which will be beneficial for studying the molecular mechanisms of wood formation.

## 1 Introduction

Programmed cell death (PCD) is a highly regulated program in cell differentiation, and it widely exists in various biological processes. In most multicellular organisms, PCD is part of normal development and growth processes. Wood formation is a typical PCD process (Courtois et al., 2009; Jiang et al., 2021; Cao et al., 2022). The secondary xylem cells are derived from the vascular cambium, and the matured xylem includes xylem fiber cells with thick walls for mechanical support, vessel cells for vertical water transport, and ray cells for radial transport and storage of nutrients. One of the major developmental events of xylem cell differentiation is the periclinal divisions of cambial meristem cells to produce xylem mother cells (Du et al., 2023). Xylem mother cells then undergo differentiation processes including cell elongation and expansion, secondary wall thickening and lignin deposition, PCD, and final autolysis to form matured secondary xylem cells (Du and Groover, 2010; He et al., 2022). After the concept of PCD was introduced into plant developmental biology, many studies have proved that the differentiation process of xylem tracheary element (TE) in woody plant is a typical PCD process (Pyo et al., 2007; Escamez and Tuominen, 2014).

Previous research had introduced several traditional methods to identify and quantify PCD progress in woody plants, such as microscopic observation, DNA ladder assay, and TUNEL detection. (Majtnerová and Roušar, 2018). However, microscopic examination can only provide the information of morphological changes of PCD cells. Although DNA ladder assay could determine the “DNA ladder” pattern of DNA fragments that occurred during the PCD progress, it can only be used for detecting PCD at a later stage when it is believed to have been ongoing (Majtnerová and Roušar, 2018). As for TUNEL, the *in situ* labeling method for detecting fragmented nuclear DNA to label the PCD process (Moore et al., 2021), there are also several shortcomings that are hard to overcome. First, although TUNEL is mostly used to detect cells with early apoptosis based on the DNA breakage that occurs in the early stage of apoptosis, false positive signals from necrotic cells cannot be totally avoided (Gorczyca et al., 1993). Meanwhile, DNA damage from other sources can also cause false-positive TUNEL signals. In addition, considering that TUNEL detection consists of many steps including tissue fixation, embedding, sectioning, and TUNEL reaction, the incorrect operation of any single step could also lead to false signals. Therefore, new methods need to be developed for the study of the PCD process in woody plants.

Flow cytometry, which allows the analysis of cells in suspension, has been developed in the identification and quantification of PCD (Weir et al., 2005). The cell shape, size, and granularity changes during the PCD process can be inferred from forward scatter (FSC) and side scatter (SSC). The FSC relates to the cell diameter, while the SSC relates to the inner cellular structures (McKinnon, 2018). The advantage of this method is the possibility of combining the scatter signals with fluorescence to allow for the identification of subgroup cells undergoing PCD. In the early stage of PCD, alterations occur at the cell surface: one of the alterations is the translocation of phosphatidylserine (PS) from the inner side of the plasma membrane to the outer layer, by which PS is exposed at the external surface of the cell (Dwyer et al., 2012). At this stage, cells became annexin V positive. In the late stage of PCD, the cell plasma membrane has been compromised, and the cell nuclei could be stained with PI (Nicoletti et al., 1991). Therefore, PI is widely used in conjunction with annexin V to determine if the cells undergoing PCD are in the early or late PCD process (Rieger et al., 2011). Accordingly, it is possible to use flow cytometry to sort cells at different stages during the PCD process based on fluorescence-activated cell sorting (FACS).

FACS is a powerful technique that is widely used for the isolation of cells based on fluorescence. Thousands of cells can be quantified and collected within just a couple of minutes, and different populations can be harvested simultaneously (Clark et al., 2022). FACS has been effectively used to characterize the transcriptome of *Arabidopsis* root cells (Birnbaum et al., 2005) and successfully used to isolate maize root endodermal cells (Ortiz-Ramírez et al., 2018). Recently, self-transcribing active regulatory region sequencing and transcriptomic analysis were performed in sorted cells using FACS in *Arabidopsis* and rice (Rich-Griffin et al., 2020; Tian et al., 2022). However, it is rare to detect gene expression alterations during the PCD process by using FACS in woody plants. The diversity of secondary cell wall thickness increases the difficulty of isolating viable cells in woody plants.

**Figure 1:**
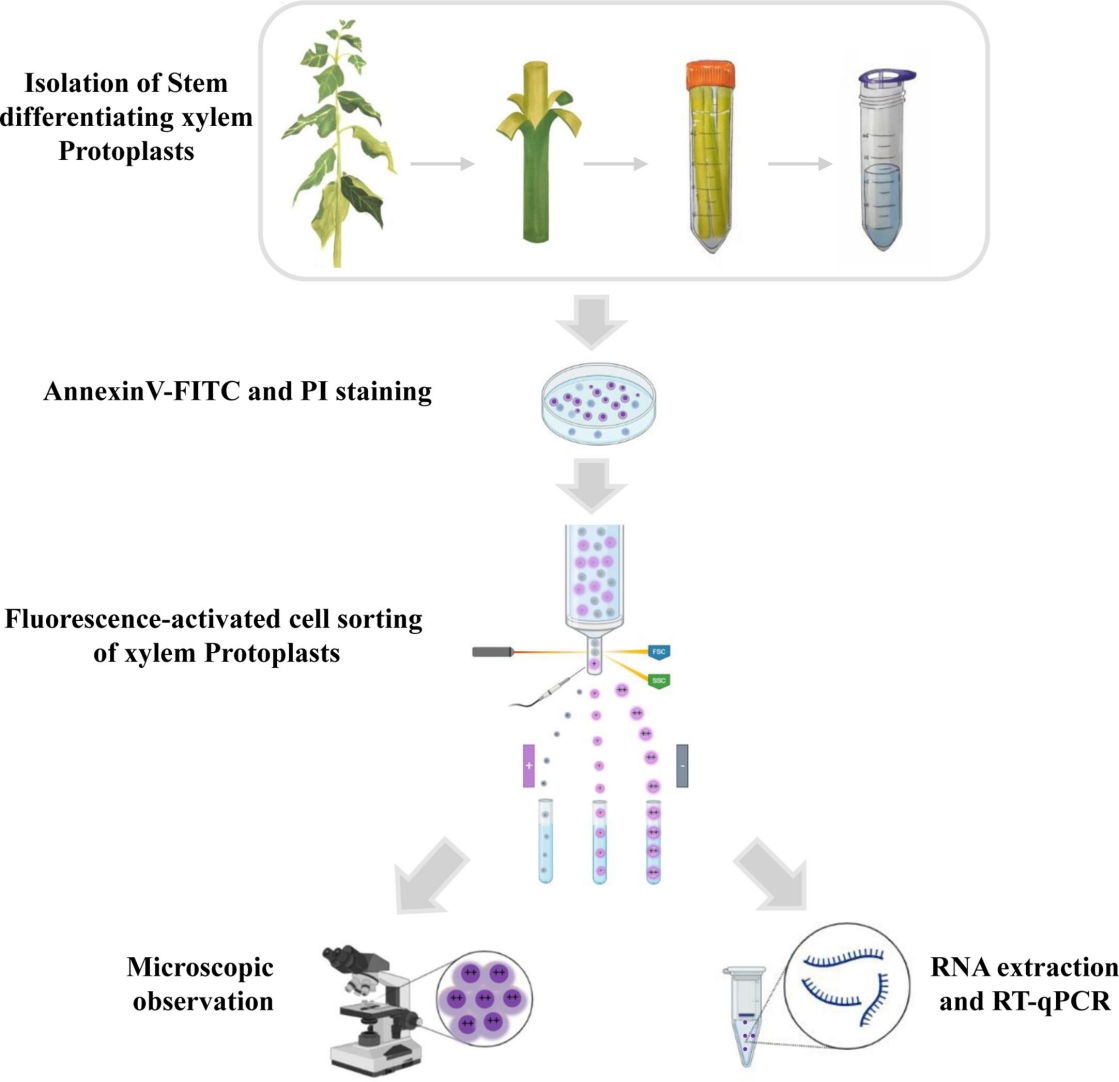
Schematic overview of the experiment for programmed cell death in xylem protoplasts using fluorescence-activated cell sorting method. The stems of the poplar were peeled off the bark and digested in an enzymatic solution to remove the cell walls. Protoplasts were then filtered through a cell strainer and collected for downstream analysis. The isolated protoplasts were stained with annexin V-FITC and PI. During FACS, cells can be separated based on different features such as the presence of a fluorophore, cell size, and cell shape. After sorting, different subpopulations of xylem cells were observed by microscope, and the RNA was isolated for transcription research.

Here, we developed a new, simple, and reproducible method that uses developed and implemented flow cytometric techniques combined with microscopic observations and quantitative reverse transcription polymerase chain reaction (qRT-PCR) to analyze the cells in different stages of PCD in woody plants. The workflow is portrayed in **Figure 1**.

## 2 Materials and methods

### 2.1 Plant materials

The hybrid poplar *Populus alba×P. glandulosa* ‘84K’ was used in this study. The plants were grown in a greenhouse for three months with long-day conditions (16 h light/8 h dark) at 25°C and with a light intensity of 80 mmol m^-2^ s^-1^ and 60% humidity.

### 2.2 Isolation of stem-differentiating xylem protoplasts

The isolation of protoplasts from stem-differentiating xylem was carried out as previously described (Lin et al., 2014; Liu et al., 2021) with minor modifications. Briefly, the stem below the 5^th^ internode was separated into bark and wood and then cut into about 10 cm-long segments. The segments from wood tissues were submerged in freshly prepared cell wall digestion enzyme solution [1.5% (wt/vol) Cellulase R-10 (Yakult Pharmaceutical Industry, Japan) and 0.4% (wt/vol) pectolyase Y-23 (Yakult Pharmaceutical Industry, Japan) in 20 mM MES pH 5.7, 0.6 M mannitol, and 20 mM KCl solution, as well as 10 mM CaCl_2_ and 0.1% (wt/vol) bovine serum albumin] for 40 minutes in the dark at room temperature. Next, the enzyme-digested stem segments were transferred into W5 solution (2 mM MES PH 5.7, 125 mM CaCl_2_, 154 mM NaCl, 0.1 M glucose, and 5 mM KCl) and gently shaken to release protoplasts. Last, 40 µm filcons (BD Biosciences) were used to filter and collect protoplasts in the W5 solution.

### 2.3 Histological analyses

The stems used for protoplast isolation were sectioned before and after the enzyme was digested by using a vibratome (VT1000S; Leica, Wetzlar, Germany) with a thickness of 40 µm. Then, the sections were stained with 0.05% toluidine blue for 60 s at room temperature, rinsed three times in water, and photographed with a microscope (IX73; Olympus; Japan).

### 2.4 Annexin V-FITC/PI staining

The isolated protoplasts were washed twice with W5 solution, centrifuged at 200 g for 5 min, and then resuspended in 200 μL W5 solution. Next, 5 μL annexin V-FITC (solarbio, Beijing, China, Cat.No. CA1020) was added and incubated on ice for 30 min. After incubation, the protoplasts were resuspended in 200 μL WI (0.6 M D-mannitol, 20 mM MES, 20 mM KCl, 0.1% bovine serum albumin) with 1 μL of the 10 mg mL^−1^ PI stock solution for 5 min.

### 2.5 Imaging flow cytometry analysis

The suspended protoplasts were analyzed with the imaging flow cytometer Image Stream^X^ Mark II (Amnis). Samples were acquired at 60× magnification. Bright-field (420-480 nm), annexin V-FITC (505-560 nm), and PI (595-642 nm) channels were measured, and at least 10,000 events of single cells per sample were collected. Single-color compensation controls were also acquired. Image analysis was performed with IDEAS version 6.0.

### 2.6 FACS of xylem protoplasts

The suspended protoplasts were cytometrically analyzed and sorted using a BD Aria SORP cell sorter (BD Biosciences, USA). The procedure and settings used were as in previous reports (Carqueijeiro et al., 2016). A 100 µm size nozzle and 20 psi sheath pressure were used with flow rates of 5,000 events/s. FSC and SSC were used for gating protoplasts from debris. FITC and PI fluorescence were set to separate negative, early PCD, and late PCD xylem cells, and 10,000 events were displayed for each plot.

### 2.7 Microscopic observation of sorted cells

After sorting, different subgroups of xylem cells were centrifuged at 200 g for 5 min. The supernatant was removed, and 10 µL suspension was evenly coated on the slide. All microscope observations and image acquisitions were performed using the Olympus IX73 (Japan) with 20× magnification. The cells before sorting were also photographed to observe the cell morphology.

### 2.8 RNA extraction and cDNA synthesis

Sorted cells were collected in microcentrifuge tubes containing RNA extraction buffer RTL with 2-mercaptoethanol. The total RNAs were extracted from cells from the Q2, Q3, and Q4 gates and referred to the method of Birnbaum (Birnbaum et al., 2005). The RNA was isolated using a RNeasy micro extraction kit and RNase-free DNase I set (Qiagen, Hilden, 74004, Germany) with some modifications (adjusting the RTL buffer to 3.5 times of the cell suspension and adjusting 2-mercaptoethanol to 10 µL per 1 mL RLT buffer). First-strand cDNA synthesis was carried out with approximately 300 ng RNA using the SuperScrip III first-strand synthesis system (TaKaRa, Dalian, RR047A, China) according to the manufacturer’s instructions.

### 2.9 qRT-PCR

The genes related to the PCD process and vessel, fiber, ray, and cambium development were selected. Specific qRT-PCR primers were designed with melting temperatures of 58-60°C and amplicon lengths of 80-150 bp using Primer3 software (https://bioinfo.ut.ee/primer3-0.4.0/); these are given in **Supplementary Table S1**. qRT-PCR was performed in quadruplicate using the TB Green^®^ Premix EX Taq ™ II (TaKaRa, Dalian, RR820A, China) on a Roche lightCycler 480 (Roche Applied Science, Penzberg, Upper Bavaria, Germany) according to the manufacturer’s instructions. The expression level of the genes was normalized to that of *PagActin* using Roche LightCycler advanced relative quantification analysis. The qPCR program started with a 30 s initial denaturation step at 95°C, followed by 40 cycles of amplification (95°C for 5 s, 60°C for 30 s) with continuous monitoring of the SYBR Green fluorescence. The reaction ended with a melting curve step from 55°C to 95°C at 0.5°C per second. All experiments were repeated at least three times with similar results.

## 3 Results

### 3.1 Harvesting protoplasts from differentiating xylem

**Figure 2:**
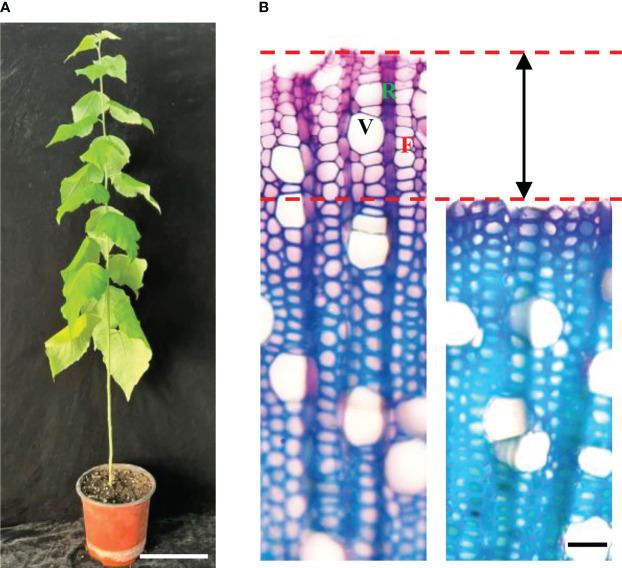
Differentiating xylem (DX) protoplast preparation. **(A)** Three-month-old poplar. (bar = 10 cm). **(B)** Cross-sections view of stems showing that about 10 layers of DX cells were released for the protoplast preparation. Left: before enzyme digestion; right: after enzyme digestion (bar = 20 µm). F, fiber; R, ray cell; V, vessel.

**Figure 3:**
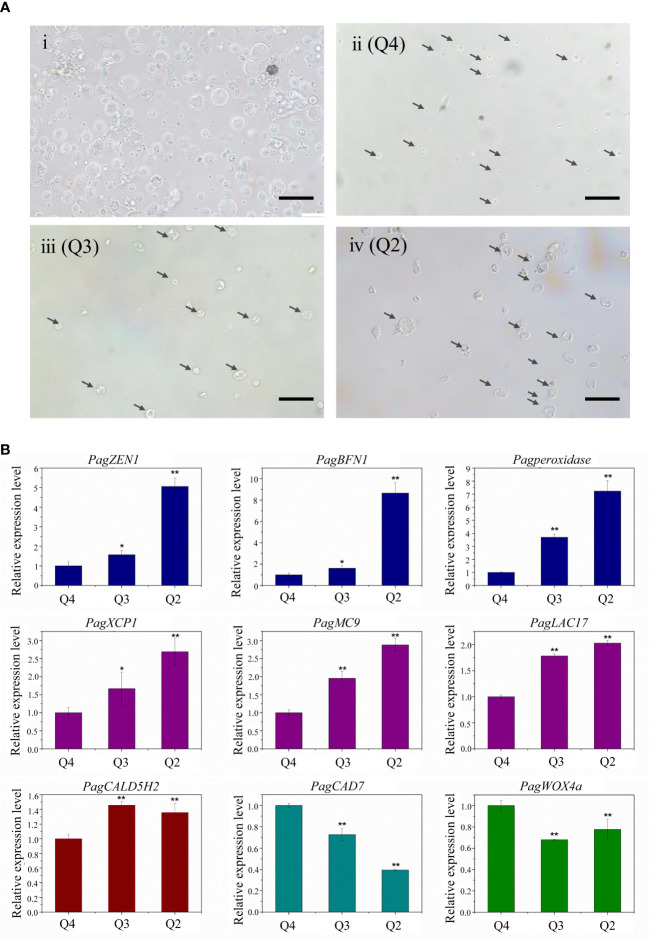
**(A)** Microscopic observation of different stages of xylem protoplast. (Ai) Xylem protoplast before fluorescence-activated cell sorting. (Aii) Annexin V-FITC and PI double negative healthy cells. (Aiii) Annexin V-FITC positive PCD cells. (Aiv) Annexin V-FITC and PI double positive PCD cells. (bar = 20 µm). **(B)** Expression levels of PCD and wood formation-related genes. Student’s t-test; *P < 0.05; **P < 0.01.

To isolate protoplasts from hybrid poplar 84K, we used stems harvested from six 3-month-old 84K plantlets (**Figure 2A**). We then separated the bark and wood tissues of stems below the 5^th^ internode and obtained free protoplasts from the differentiating xylem using a previously described digestion method (Lin et al., 2014; Liu et al., 2021). Stem sectioning showed that about 10 layers of differentiating xylem cells were released by enzyme digestion (**Figure 2B**). Most cells in the differentiating vascular tissue had been digested and released. The collected protoplasts were derived from differentiating fiber, vessel, and ray cells, which are the three major cell types that form wood (Tung et al., 2023), and they may also contain a small amount of cambium cells. The photograph of protoplasts harvested from differentiating xylem showed that the cell shape was round, and the cell membrane was complete, indicating that the cell vitality was good. The size of the protoplast cells was diverse, with cell diameters ranging from about 2 μm to 20 μm (**Figure 3Ai**).

### 3.2 Imaging flow cytometry-based analysis of xylem PCD cells

**Figure 4:**
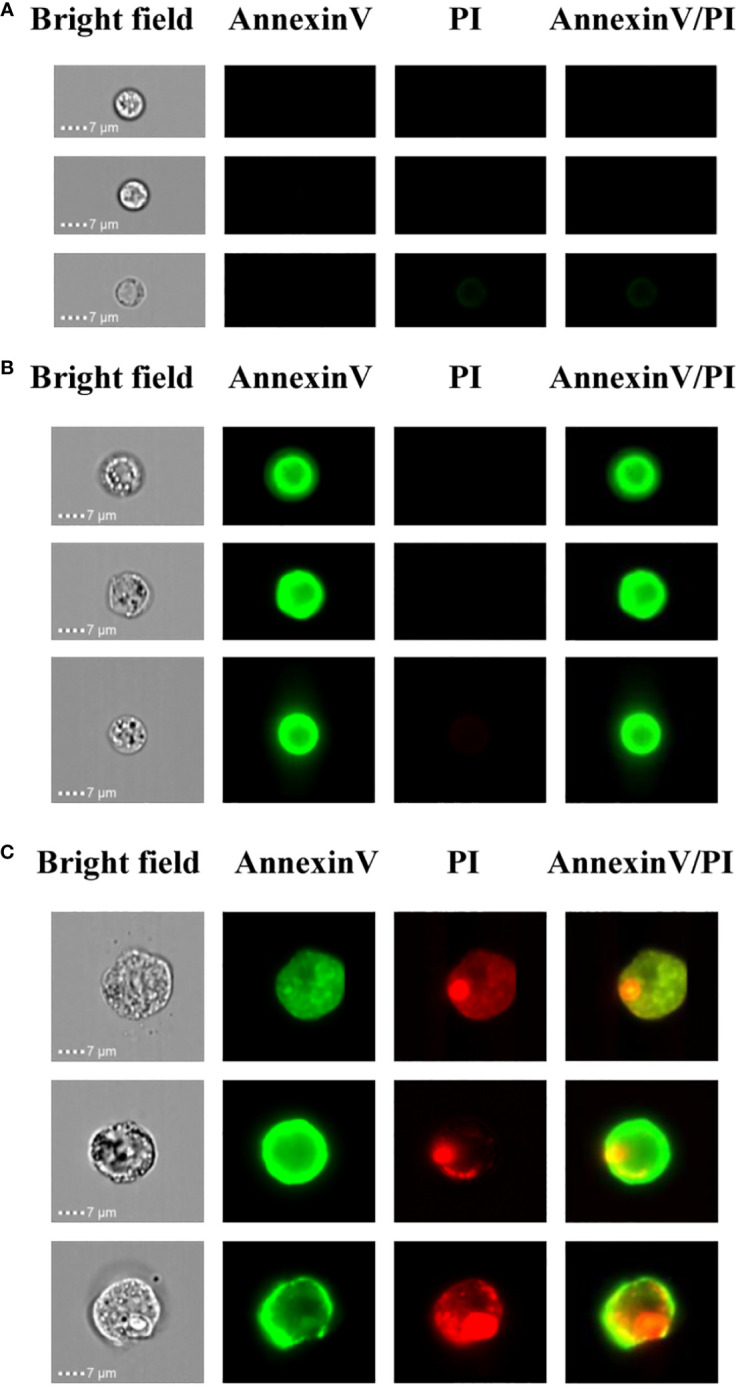
Imaging flow cytometry-based analysis of xylem PCD cells. **(A)** Annexin V-FITC and PI double negative healthy cells. **(B)** Annexin V-FITC positive and PI negative PCD cells. **(C)** Annexin V-FITC and PI double positive PCD cells.

In order to determine the PCD of xylem cells based on cell size and fluorescence intensity, we employed the imaging flow cytometer for cell detection. Here, imaging flow cytometry analysis was carried out after annexin V-FITC and PI staining of the xylem cells. On the image stream, each cell had four images, namely, BF (bright field, channel 1), annexin V-FITC (false green, channel 2), PI (false red, channel 3), and annexin V-FITC/PI (channel 4). Cells from the first subgroup had a round morphology observed in the bright-field channel. In addition, they could be categorized as double negative because neither annexin V-FITC nor PI staining could be detected. The cell size of this subgroup was around 1-7 μm, and the cell membrane was complete, indicating that their cell activity was good (**Figure 4A**). Cells from the second subgroup appeared slightly shrunken with more complex morphologies under bright fields than cells from the first subgroup. Furthermore, annexin V-FITC staining could be detected in cells from this subgroup, indicating phosphatidylserine exposure. However, the PI signals of cells from this subgroup were negative. The cell size of this subgroup was about 8-10 μm. The annexin V-FITC positive and PI negative character of this subgroup suggest the cells were in the early PCD stage since those cells had intact membranes preventing PI staining (**Figure 4B**). Cells in the third subgroup were the largest, irregularly shaped with condensed, fragmented nuclei. The cell size of this subgroup was about 15 μm. Due to the membrane damage, PI could penetrate into the cells and thereby be detected, resulting in the cell membrane possessing a green FITC fluorescence signal, and the nucleus had a red PI fluorescence signal. Thus, these cells were double positive (**Figure 4C**). According to the staining results, it is speculated that these group cells were undergoing late PCD.

### 3.3 FACS and microscopic observation in different PCD stages

**Figure 5:**
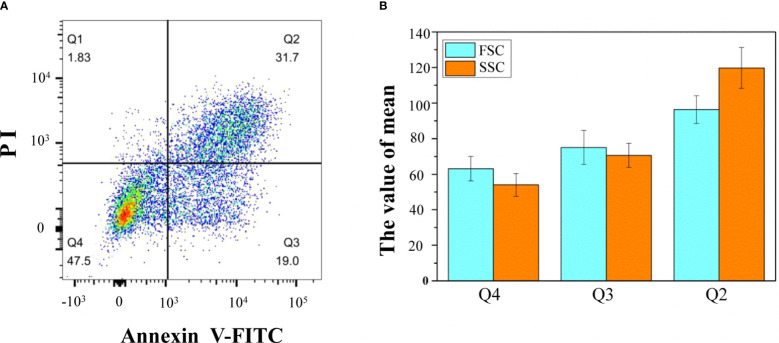
Flow cytometric analysis of annexin V-FITC/PI staining of differentiating xylem protoplast. **(A)** The upper-left quadrants (Q1) contain the annexin V-FITC negative and PI positive cells; the upper-right quadrants (Q2) contain the annexin V-FITC positive and PI positive cells; the lower-right quadrants (Q3) contain the annexin V-FITC positive and PI negative cells; the lower-left quadrants (Q4) contain the annexin V-FITC negative and PI negative cells; **(B)** the mean values of different types of cells. One representative experiment out of three is shown.

By staining isolated protoplasts with annexin V-FITC and PI, fluorescence-activated cell sorting (FACS) was used to distinguish and quantitatively determine the percentage of cells during different stages of the PCD process (**Figure 5A**). The whole-cell group was displayed in a dot plot with annexin V-FITC as abscissa and PI as ordinate. The Q1 gate (annexin V-FITC negative and PI positive, representing necrosis cells not undergoing PCD) accounted for 1.83%, which can be ignored. The Q2 gate annexin V-FITC positive and PI positive cells, accounting for 31.7% of the total cells, were judged as late PCD cells. The Q3 gate annexin V-FITC positive and PI negative cells, accounting for 19% of the total cells, were judged as early PCD cells. The Q4 gate annexin V-FITC negative and PI negative cells, accounting for 47.5% of the total cells, were judged as healthy cells. The FSC and SSC signals were used to measure cell size and complexity, respectively. A higher FSC signal represents apparently larger cells. A higher SSC signal means higher complexity as a consequence of cellular changes. In this study, according to the mean values of FSC and SSC, the healthy cells were the smallest, and the late apoptotic cells were the largest (**Figure 5B**).

Microscopic observation allows the visualization of biochemical and molecular changes associated with the PCD process and distinguishing morphologic changes of cells in different PCD stages. The microscopic observation of xylem protoplast cells after FACS showed that the morphological differences among different subgroups were distinct. The cells from Q4 were small and round, and its plasma membrane was integrity, which confirmed that these cells are healthy (**Figure 3Aii**). Accordingly, the cells of this subgroup corresponded to the annexin V-FITC negative/PI negative subgroup, as determined by imaging flow cytometry, which could be referred to as healthy cells. The cells from Q3, which correlated with the early PCD stage, were slightly larger. Although the cells shrank, their membrane remained intact, which is consistent with the characteristics of cells during the initial stages of PCD (**Figure 3Aiii**). These cells corresponded to the cells in the annexin V-FITC positive/PI negative subgroup detected in the imaging flow cytometry analysis. As for Q2, the cells were the largest, and an irregular shape with shrinkage, plasma membrane blebbing, cell detachment, and nuclear condensation were observed, indicating these cells were in the late PCD stage (**Figure 3Aiv**). These cells had the character of cells in the late PCD process and were consistent with the cells in the annexin V-FITC positive/PI positive subgroup in the imaging flow cytometry analysis. The fine consistency of the data from FACS and the image stream fluorescence analysis indicates that using cell imagery or the morphology of gated populations can clearly distinguish xylem cells before or in the early and late stage of the PCD process in woody plants.

### 3.4 Analysis of the expression of marker genes in different PCD stages

To test the feasibility of using the sorted cells for transcription studies, the sorted xylem cells were collected directly for RNA extraction. RNA from the Q2, Q3, and Q4 subgroups was isolated separately, with a yield of 300 ng of RNA from about 50,000 cells. qRT-PCR analysis of the genes involved in the PCD process and xylem development was carried out (**Figure 3B**). The expression of PCD-related genes (*PagZEN1*, *PagBFN1*, *PagPeroxidase*) showed the lowest expression in the Q4 subgroup and the highest in Q2 subgroup, corresponding well with the living and intact cells and cells in the late PCD process. During wood formation, the expression of genes related to vessel development (*PagXCP1*, *PagMC9*, *PagLAC17*) also increased with the occurrence of PCD. The fiber development-related gene *PagCAld5H2* was highly expressed in the Q3 subgroup, indicating early PCD. The expression of the ray parenchyma development-related *PagCAD7* gene was higher in the Q4 subgroup. The highest expression of the cambium development-related gene *PagWOX4a* was also found in the Q4 subgroup, which involved most living cells, indicating that there was a small number of cambium cells in the stripped stem segments.

## 4 Discussion

Secondary xylem is the most abundant biomass produced by land plants and is important for global carbon sinks (Ye and Zhong, 2015). After the deposition of secondary walls, TEs and fibers undergo PCD, resulting in the degradation of their protoplasts (Escamez and Tuominen, 2014). The lack of efficient and accurate methods to detect PCD in woody plants has seriously hindered the progress of basic research in this field. Flow cytometry is a technique that enables the analysis of the population dynamics of cell development, the measurement of cell size and shape, and even the fluorescence characteristics of suitable labeled single cells (Weir et al., 2005). Here, we used flow cytometry to investigate each developmental stage in the developmental process during the PCD of xylem cell in woody plants.

The isolation of protoplasts by tissue digestion and cell wall removal without damaging the cell membrane was the crucial step for FACS analysis in the woody plant. In order to obtain a protoplast with good quality for xylem cell sorting, an optimized protocol was developed to digest cells for the production of a high yield of pure and stable xylem protoplasts from wood-forming tissues. After digestion, the isolated xylem protoplasts are generally larger in diameter (**Figure 4**, **Figure 5A**) than the mammalian cells (Antoniadi et al., 2022), which are osmosensitive and fragile, so the concentration of mannitol in the collected liquid was adjusted to keep the integrity of protoplasts. The obtained protoplasts involved fiber, vessel, and ray cells, three major cell types that form wood. They also contained a small number of cambium cells (**Figure 2B**). These different kinds of cells were successfully separated by FACS in our experiment (**Figure 5**).

To obtain xylem cells at specific developmental stages from wood-forming tissues for transcriptional studies, stained cells were isolated using FACS, and the total RNA was isolated for qRT-PCR analysis. Vessel elements are a specific type of TE that die after secondary cell wall thickening. ZEN1 is a key enzyme in the degradation of nuclear DNA during the PCD of TEs (Ito and Fukuda, 2002). *BEN1* encodes S1-type DNases that are associated with PCD in plants (Farage et al., 2011). In our results, the expression of *PagZEN1* and *PagBFN1* was the highest in the Q2 subgroup, indicating these cells were in the late PCD process. *Arabidopsis* xylem cysteine proteases XCPs were reported to accumulate in the vacuole and work in PCD-associated cell clearance during TE differentiation. *XCP1* was frequently used as the marker for xylogenesis and TE PCD and was specifically expressed in the vessel element (Avci et al., 2008; Liu et al., 2020). *PagXCP1* and *PagLAC17* were shown to be expressed at the later stage PCD of vessel formation in cluster 7 (Li et al., 2021), which was consistent with our results in this study. *AtMC9* is a xylem-specific metacaspase located in the apoplast and vacuole, and it has been shown to participate in regulating TE post-mortem autolysis (Bollhöner et al., 2013) and xylem cell death (Courtois et al., 2009). The expression of *PagMC9* was also increased with the occurrence of PCD. *CAld5H2* was identified as the fiber-specific marker in *P. trichocarpa* by laser microdissection (Shi et al., 2017), which was significantly upregulated in cluster 2 and involved in the S monolignol biosynthesis in fiber cells (Li et al., 2021). Our results showed that the expression of *PagCAld5H2* was more upregulated in Q3 than in Q2. *PagCAD7* was shown to be localized in the xylem parenchyma cells, providing lignin precursors to the adjacent vessels and fibers (Chen et al., 2021; Li et al., 2021). In our study, we detected that the expression of the *PagCAD7* gene was the highest in the healthy cell subgroups. qRT-PCR analysis of the genes involved in the PCD and different types of xylem cells further confirmed the suitability of the sorted cells for transcriptomic studies, which will benefit the study of transcriptional profiles from specific cell populations in woody plants.

We used imaging flow cytometry for the PCD research of woody plants for the first time. Imaging flow cytometry provides unique opportunities in the detecting and preparing images of individual cells that are being analyzed. The combination of microscopy and flow cytometry makes it possible to take images of xylem cells in bright fields and different fluorescence channels. It can obtain the images of several thousand cells with 20× magnification in a short period of time (Pietkiewicz et al., 2015). This study demonstrated the ability of the image stream flow cytometry to discriminate healthy cells and the early and late stages of PCD xylem cells. The developing xylem cells contain different types of cells, such as vessels, fibers, rays, which have distinct cell sizes. Combined with fluorescent staining at different stages of apoptosis, imaging flow cytometry can effectively distinguish them, which will be helpful for observations and statistics analyses of the PCD of different types of xylem cells in woody plants.

According to the analysis of the expression level of xylem developing related cell-type-specific marker genes, the cell morphology, and the imaging flow cytometry observation of the sorted cells, it could be speculated that the healthy cell subpopulations (Q4) may have included cambium cells, ray cells, and some fiber cells that had not undergone PCD. The subpopulations of the early PCD cells (Q3) were composed mostly of fiber cells. The subpopulations of late PCD cells (Q2) were composed mostly of vessel cells. This combination of classical flow cytometry with the microscopy method makes it possible to analyze the biochemical and morphological features of dying cells directly in one measurement, thereby allowing for the quantification of several thousand cells in a short period of time, together with an objective analysis of pictures by image-based features (George et al., 2004). Additionally, the staining procedure of this method is simple, and the results could be rapidly obtained and reproducible. Notably, this method facilitates the identification of gene expression levels for specific cells at different stages of PCD.

Flow cytometer in combination with the classical annexin V/PI staining allowed us to establish the method for distinguishing the early and late stage of xylem cells during PCD at the single-cell level. Compared to previous PCD determination by TUNEL at the organ level, our method exerts a better resolution to explore the xylem cells in the specific developmental process of PCD. Meanwhile, this method is not limited to poplar trees but is applicable to other woody plant materials. In addition, this method can also be used for the function characterization of PCD-related genes in transgenic materials.

## 5 Conclusion

We have developed a method using flow cytometry to rapidly analyze cells undergoing the PCD process and using FACS to sort different kinds of PCD cells in woody plants. This method was carried out using isolated xylem cells with annexin V-FITC and PI staining to reveal the PCD process. The cell morphology observed by microscopy and the expression level of cell-type-specific marker genes determined by qRT-PCR further confirmed the reliability of this method. This new method will facilitate the analysis of the PCD process of woody plants.

## Data availability statement

The original contributions presented in the study are included in the article/**Supplementary Material**. Further inquiries can be directed to the corresponding author.

## Author contributions

Y-LL: performing—the experiments and data curation; writing—original draft and editing. Y-HG: performing—the experiments and editing. X-QS: writing—reviewing and editing. M-XH: performing—the experiments. S-TZ: designing the study, writing—reviewing and editing, and funding acquisition. All authors read and approved the final manuscript.

## Supplementary material

Supplementary Table 1 List of primers used in this work.
