# Supplementary material for: A method for analyzing programmed cell death in xylem development by flow cytometry
Source: Front Plant Sci. 2023 Jun 9;14:1196618. doi: 10.3389/fpls.2023.1196618 (PMC10288846; doi:10.3389/fpls.2023.1196618)
Supplement: Supplementary Table 1 — List of primers used in this work. [file Table_1.doc]

|  | | | |
| --- | --- | --- | --- |
| Table 1 The primer pair sequences used for quantitative real-time PCR | | | |
| 引物 | 引物序列（5^，^-3^，^） | 引物 | 引物序列（5^，^-3^，^） |
| Primer | Primer sequence（5^，^-3^，^） | Primer | Primer sequence（5^，^-3^，^） |
| *PagZEN1-F* | TCCTGGAAATGGGAATTGTG | *PagZEN1-R* | TTGAGTGCATGCTCCTGTTC |
| *PagBFN1-F* | TCGTCGATATAATTTGACAGAAGC | *PagBFN1-R* | CGGTGTTTCCTCCTTCATCA |
| *PagPeroxidas -F* | TGTGATGGGTCTGTGCTGAT | *PagPeroxidas-R* | GAGACAACACCAGGGCATTG |
| *PagXCP1-F* | TCTTTGCGTCACTTTTCGTG | *PagXCP1-R* | CTCATTCAACCCAAGCCAGT |
| *PagMC9-F* | TGGAGCATTTAGCAATGCAG | *PagMC9-R* | GGCTGCCACAAGAAAGTAGC |
| *PagLAC17-F* | GCTAGACCTTACGCGACTGG | *PagLAC17-R* | TGGTGGAGTGGTTTGATTGA |
| *PagCAld5H2-F* | CGATATGGTCGATGACATGC | *PagCAld5H2-R* | GTCTCTGTCCCACCGAACAT |
| *PagCAD7-F* | CTACTCGGACATTATGGTCGTAGAT | *PagCAD7-R* | TAAAATCTCAAGGGGCTATACACTG |
| *PagWOX4a -F* | CGAACCCTCTCTTACGCTTG | *PagWOX4a -R* | GCTATCGGGTCTGATGAAGC |
| *PagActin-F* | AAACTGTAATGGTCCTCCCTCCG | *PagActin-R* | GCATCATCACAATCACTCTCCGA |
